# Effect of cumulative radiation exposure from Coronary catheterization on lung cancer mortality

**DOI:** 10.1186/s12885-023-11231-4

**Published:** 2023-08-15

**Authors:** Jin Liu, Shiqun Chen, Yang Zhou, Xueyan Zheng, Ruilin Meng, Ning Tan, Yong Liu

**Affiliations:** 1 Department of Cardiology, Guangdong Cardiovascular Institute, Guangdong Provincial People’s Hospital, Guangdong Academy of Medical Sciences, Guangzhou, China; 2 Department of Guangdong Provincial Key Laboratory of Coronary Heart Disease Prevention, Guangdong Cardiovascular Institute, Guangdong Provincial People’s Hospital, Guangdong Academy of Medical Sciences, Guangzhou, China; 3 Global Health Research Center, Guangdong Provincial People’s Hospital, Guangdong Academy of Medical Science, Guangzhou, China; 4 Institute of Control and Prevention for Chronic Non-Infective Disease, Guangdong Provincial Center for Disease Control and Prevention, Guangzhou, China; 5 The Second School of Clinical Medicine, Southern Medical University, Guangzhou, China; 6 School of Medicine, Guangdong Provincial People’s Hospital, South China University of Technology, Guangzhou, China

**Keywords:** Lung cancer mortality, Coronary catheterization, Radiation

## Abstract

**Background:**

Coronary catheterization (CC) procedure inevitably exposes patients with cardiovascular disease (CVD) to radiation, while cumulative radiation exposure may lead to higher risk of cancer.

**Methods:**

This multi-center, retrospective study was based on the CC procedure in Cardiorenal ImprovemeNt II cohort (CIN-II, NCT05050877) among five regional central tertiary teaching hospitals in China between 2007 and 2020. Patients without known cancer were stratified according to the times they received CC procedure. Baseline information from their last CC procedure was analyzed. Cox regression and Fine-Gray competing risk models were used to assess the relationship between cumulative radiation exposure from CC procedures and cancer-specific, all-cause and cardiovascular mortality.

**Results:**

Of 136,495 hospitalized survivors without cancer at baseline (mean age: 62.3 ± 11.1 years, 30.9% female), 116,992 patients (85.7%) underwent CC procedure once, 15,184 patients (11.1%) on twice, and 4,319 patients (3.2%) underwent CC procedure more than three times. During the median follow-up of 4.7 years (IQR: 2.5 to 7.4), totally 18,656 patients (13.7%) died after discharge, of which 617 (0.5%) died of lung cancer. Compared with the patients who underwent CC procedure once, the risk of lung cancer mortality increased significantly with the increase of the number of CC procedure (CC 2 times vs. 1 time: HR 1.42, 95% CI 1.13 to 1.78, *P* < *0.001*; CC ≥ 3 times vs. 1 time: HR 1.64, 95%CI 1.13 to 2.39, *P* < *0.05*). Similar results were observed in all-cause mortality and cardiovascular mortality, but not in other cancer-specific mortality.

**Conclusions:**

Our data suggest that substantial proportion of CVD patients are exposed to multiple high levels of low-dose ionizing radiation from CC procedure, which is associated with an increased risk of cancer mortality in this population.

**Trial registration:**

ClinicalTrials.gov: NCT05050877; URL:http://www.clinicaltrials.gov; 21/09/2021.

**Supplementary Information:**

The online version contains supplementary material available at 10.1186/s12885-023-11231-4.

## Introduction

Global Burden of Disease Study has reported that cardiovascular diseases and cancers are the two most deadly non-communicable diseases globally [1], and patients with cardiovascular disease develop or concomitant cancers can lead to a greater burden of clinical care and a worse prognosis [2–4]. To diagnosis these patients, radiological image diagnostic examination is essential, such as traditional radiography and computed tomography (CT) scans, but it is also significantly associated with higher incidence of cancer, dermatitis, cataracts, and other adverse health effects [5–8].

Coronary catheterization (CC), as the most accurate and routine examination for diagnosing cardiovascular disease clinically, have 5–10 times of radiation than CT scan or other traditional radiographies [9]. Previous studies reports cardiac imaging can increase the risk of cancer beside abdominal and pelvic, especially lung cancer [10, 11]. For patients with cardiovascular disease, the effect of radiation exposure from CC on cancer mortality is unclear. In addition, the morbidity and mortality of certain malignancies like leukemia, multiple myeloma and lymphoma increase with the elevation of radiation dose from CT scan or other conventional diagnostic X-rays [12–15]. However, for CC procedure, especially among patients with in-stent restenosis and revascularization, multiple angiographic procedures and exposure to radiation are inevitable. There is very little evidence on effect of CC procedure on lung cancer mortality, and the specific risk of different cancer mortality increased by multiple radiography is unclear.

Our aim is to assess the effect of cumulative radiation exposure from multiple CC procedures on the cancer mortality (especially lung cancer mortality) among patients without known cancer in the Cardiorenal ImprovemeNt II cohort.

## Methods

### Study population

**Fig. 1:**
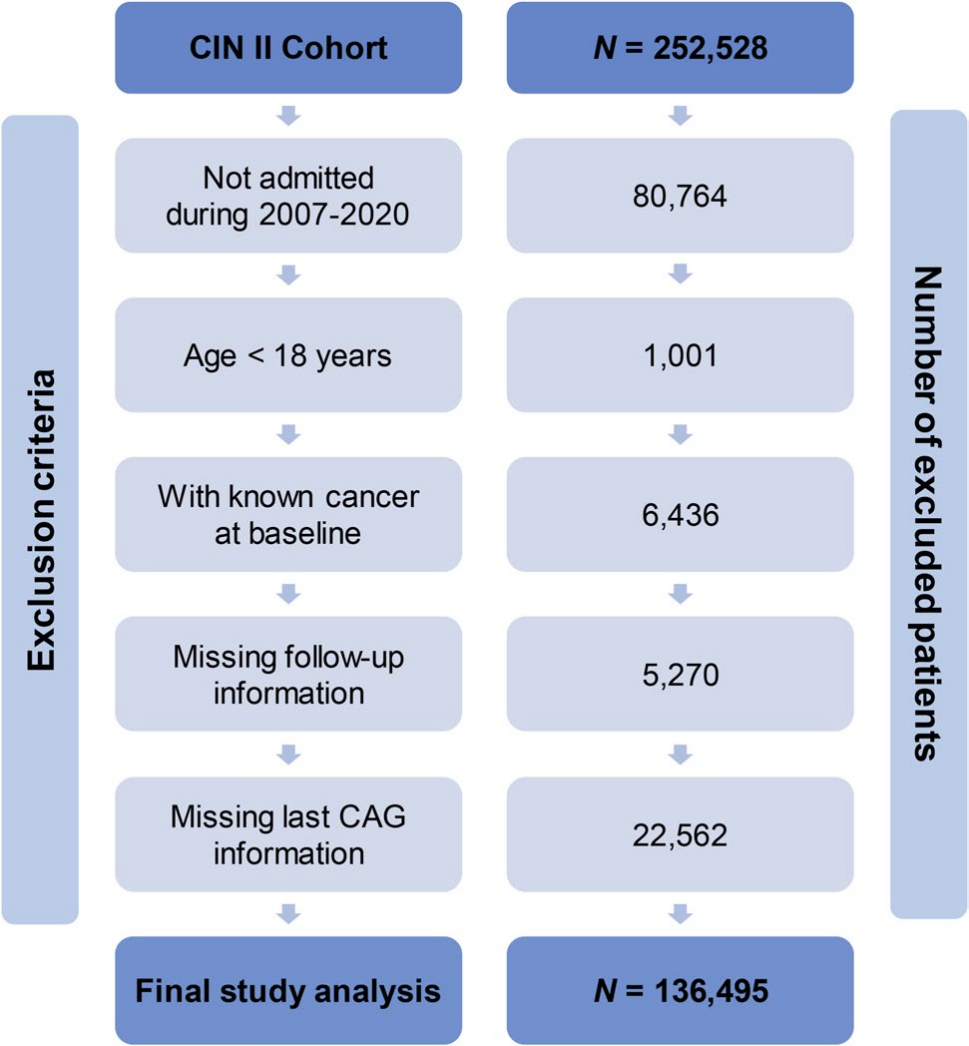
Flow diagram

This multi-center, retrospective study recruited patients undergoing coronary catheterization procedures in Cardiorenal ImprovemeNt II (CIN-II, NCT05050877, 21/09/2021) cohort among five regional central tertiary teaching hospitals in China between 2007 and 2020 [16]. Patients admitted for CC procedures (≥ 18 years) were included in this study from 2007 to 2020 and their baseline information from the last angiography were analyzed. We excluded: 1) patients with known cancer at admission; 2) patients who died during hospitalization; 3) patients missing follow-up information. Finally, 136,495 participants in CIN-II were enrolled into study analysis (Fig. 1).

The study protocol was approved by the Ethics Committee of Guangdong Provincial People’s Hospital (No.GDREC2019-555H-2), all participating sites received institutional review board approval from their own ethics committees, and the study was performed according to the declaration of Helsinki. The need for informed consent was waived because of the retrospective nature of the study.

### Data collection

In-hospital data was collected from the Electronic Clinical Management System (ECMS) for all participant hospitals. The data was mainly included six sections: demographics, discharge diagnosis, laboratory examinations, operation procedures, discharge medication, and discharge status. Further details on data governance have been published previously [16]. To identify the survival information of patients, we also linked cause-specific surveillance dataset at the regional Center for Disease Control and Prevention, by using the unique personal identity number.

Because specific dose was not routinely collected, we imputed an effective dose for each CC procedure (including coronary angiography [CAG] and percutaneous coronary intervention [PCI]) based on the typical effective dose in the literature. We then calculated the total cumulative effective doses of radiation (in milliSieverts [mSv]) from all CC procedures received by each subject during 2007–2020.

### Study outcomes and definitions

The primary outcome was lung cancer mortality, and secondary outcomes were all-cause mortality, cardiovascular mortality, other cancer-specific mortality. Cause-specific mortality was categorized by the main reason of death with International Classification of Diseases (ICD) 10^th^ Edition. Death case with missing values of ICD-10 information have been excluded from the case-specific analysis. The cardiovascular mortality was mainly identified by ICD-10 codes: I00-I99, Q20-Q28, N00-N08, N10-N16, N17-N19 [17]. Coronary artery disease (CAD) was confirmed by coronary angiography (CAG), and the main artery stenosis was more than 30% and one of the other three coronary vessels stenosis was more than 50%. Hypertension (HT) was defined according to the 10th Revision Codes of the International Classification of Diseases (I10.xxx-I12.xxx, I15.xxx and I67.400). Diabetes mellitus (DM) was defined as ICD-10 codes, the utilization of oral hypoglycemic drugs or insulin when discharged from the hospital or the glycated hemoglobin more than 6.5%. Congestive heart failure (CHF) was confirmed by the 10th Revision Codes of the International Classification of Diseases (ICD-10) for congestive heart failure (I50.001, I11.000, I13.000, I50.907 and R57.000 etc.) and the heart function classification of the New York Heart Association (NYHA) class > 2 or Killip class > 1 [18]. Chronic kidney disease (CKD) was defined as the discharge diagnosis and eGFR ≤ 60 mL/min/1.73 m [2] using the Chronic Kidney Diseases Epidemiology Collaboration equation (CKD-EPI) [19]. Anemia was defined using World Health Organization criteria: baseline hematocrit value < 39% for men and < 36% for women [20].

### Statistical analysis

We described demographics, treatments and outcomes of patients receiving CC procedures. We divided the patients into three groups according to the number of their CC procedures: patients receiving CC for 1 time, patients receiving CC for 2 times, and patients receiving CC for ≥ 3 times, and evaluated whether the most radiation-exposed patients were at increased risk of cancer. Additionally, we also assessed the association of high radiation exposure (> 15 mSv) relative to low exposure (≤ 15 mSv) with the risk of cancer [11, 21, 22]. The categorical variables were presented as numbers (percentage), and continuous variables were presented as means (SD) or median [interquartile ranges (IQR)]. Chi-Square test, Kruskal-Wallis test and ANOVA test were used to compare the differences between the groups as appropriate. Tukey and Kramer (Nemenyi) test were used to for pairwise-comparison.

Kaplan-Meier curve was used to assess the long-term all-cause mortality and compare the difference of cumulative incidence. Cumulative Incidence Function (CIF) was used to assess the cancer-specific mortality and cardiovascular mortality. Proportional hazards assumption tests were conducted for the number of angiography and the radiology dose of angiography. The association between CC procedures and radiation exposure with long-term all-cause mortality was estimated by Cox regression models. Fine-Gray competing risks models were used to assess the risk of cancer mortality and cardiovascular mortality between CC procedures and radiation exposure. Variables based on with significant baseline differences or clinical significance were included in the multivariable models (age, sex, smoking history, CAD, HT, DM, CHF, CKD, pulmonary infection, chronic obstructive pulmonary disease, atrial fibrillation [AF], stroke, anemia, high density lipoprotein cholesterol [HDLC], and low density lipoprotein cholesterol [LDLC]). To further verify the reliability of the analysis, multiple interpolation was adopted [23–25]. All analyses were performed again after imputating data based on multivariate regression variables (Analyses before imputating were showed in Supplement Table 1). In addition, the hazard ratio (HR), 95% confidence interval (CI) and P value were calculated, and variables with a missing rate of more than 20% were excluded in multivariate analysis.

Subgroup analyses were also performed to explore the source of heterogeneity according to age (≥ 65 and < 65 years), sex (male and female) and CAD (with and without). Stratification and interaction analyses showed consistency with the main results. All statistical analyses were performed using R version 4.1.1 software. A two-sided *P*-value < 0.05 indicated significance for all analyses.

## Results

### Characteristics of patients undergoing CC procedures

Our study population included 136,495 hospitalized survivors with CC procedures (mean age: 62.3 ± 11.1 years, 30.9% female). Totally 116,992 patients (85.7%) underwent CC procedures once, 15,184 patients (11.1%) underwent CC procedures procedure twice, and 4,319 patients (3.2%) underwent CC procedures for three times or more. The median radiation dose received by patients in each group were 7.0 [7.0, 15.0] mSv, 22.0 [22.0, 30.0] mSv and 37.0 [29.0, 45.0] mSv, respectively.

There were 96,430 patients (70.6%) with CAD, 70,512 patients (51.7%) with HT, 41,972 patients (30.7%) with DM, 19,898 patients (14.6%) with CHF, and 23,094 patients (18.5%) with CKD. Compared with patients receiving single CC procedure, patients undergoing multiple CC procedures had a higher rate of comorbidities including CAD, HT, DM, CKD, stroke, and anemia. More detailed information was showed in Table 1.

**Table 1:** Demographic and clinical characteristics of patients at the time of their last coronary catheterization from 2007 to 2020

Characteristics	Overall	CC = 1 time	CC = 2 times	CC ≥ 3 times	*P value*	*P value CC 1 vs 2*	*P value* *CC 1 vs* ≥ *3*	*P value* *CC 2 vs* ≥ *3*
	**136,495**	**116,992**	**15,184**	**4,319**				
**Demographic characteristics**								
Age, years	62.3 (11.1)	61.9 (11.2)	64.5 (10.6)	66.5 (10.5)	< 0.001	< 0.001	< 0.001	< 0.001
Women	42,181 (30.9)	37,938 (32.4)	3450 (22.7)	793 (18.4)	< 0.001	< 0.001	< 0.001	< 0.001
Current smoking	19,681 (20.6)	17,618 (21.5)	1628 (15.7)	435 (13.3)	< 0.001	< 0.001	< 0.001	0.001
Ex-smoking	9351 (9.8)	7516 (9.2)	1337 (12.9)	498 (15.2)	< 0.001	< 0.001	< 0.001	0.001
**Comorbidities, n (%)**								
Coronary artery disease	96,430 (70.6)	78,044 (66.7)	14,150 (93.2)	4236 (98.1)	< 0.001	< 0.001	< 0.001	< 0.001
Hypertension	70,512 (51.7)	58,669 (50.1)	9044 (59.6)	2799 (64.8)	< 0.001	< 0.001	< 0.001	< 0.001
Diabetes mellitus	41,972 (30.7)	34,057 (29.1)	5913 (38.9)	2002 (46.4)	< 0.001	< 0.001	< 0.001	< 0.001
Congestive heart failure	19,898 (14.6)	17,563 (15.0)	1779 (11.7)	556 (12.9)	< 0.001	< 0.001	< 0.001	0.042
Chronic kidney disease	23,094 (18.5)	18,706 (17.4)	3324 (24.0)	1064 (27.7)	< 0.001	< 0.001	< 0.001	< 0.001
Pulmonary infection	4939 (3.6)	4554 (3.9)	277 (1.8)	108 (2.5)	< 0.001	< 0.001	< 0.001	0.006
COPD	3886 (2.8)	3363 (2.9)	391 (2.6)	132 (3.1)	0.082	0.121	0.513	0.143
Atrial fibrillation	9803 (7.2)	8704 (7.4)	804 (5.3)	295 (6.8)	< 0.001	< 0.001	0.141	< 0.001
Stroke	8000 (5.9)	6781 (5.8)	944 (6.2)	275 (6.4)	0.040	0.112	0.185	0.753
Hyperlipemia	72,170 (52.9)	61,903 (52.9)	7954 (52.4)	2313 (53.6)	0.310	0.355	0.415	0.355
Anemia	35,606 (29.3)	29,562 (28.3)	4698 (34.7)	1346 (35.6)	< 0.001	< 0.001	< 0.001	0.281
**Laboratory tests**								
Radiation dose, mSv	15.0 [7.0, 15.0]	7.0 [7.0, 15.0]	22.0 [22.0, 30.0]	37.0 [29.0, 45.0]	< 0.001	< 0.001	< 0.001	< 0.001
Contrast medium volume, mL	106.4 (101.3)	105.5 (105.0)	112.4 (77.8)	108.4 (75.8)	< 0.001	< 0.001	0.206	0.086
eGFR, mL/min/1.73m^2^	79.9 (25.9)	80.6 (25.2)	76.3 (28.5)	74.3 (31.8)	< 0.001	< 0.001	< 0.001	< 0.001
Hemoglobin, g/L	133.9 (17.4)	134.2 (17.4)	132.3 (17.5)	132.3 (18.6)	< 0.001	< 0.001	< 0.001	0.99
Prior SCr, umol/L	0.9 [0.8, 1.1]	0.9 [0.8, 1.1]	1.0 [0.8, 1.2]	1.0 [0.8, 1.2]	< 0.001	< 0.001	< 0.001	< 0.001
LDLC, mmol/L	2.8 (1.0)	2.9 (1.0)	2.5 (0.9)	2.5 (0.9)	< 0.001	< 0.001	< 0.001	0.901
HDLC, mmol/L	1.1 (0.3)	1.1 (0.3)	1.0 (0.3)	1.0 (0.3)	< 0.001	< 0.001	< 0.001	0.068
hs-TnT, ng/L	11.7 [6.9, 30.0]	11.4 [6.7, 32.1]	12.4 [7.9, 24.0]	13.5 [8.5, 27.2]	< 0.001	< 0.001	< 0.001	< 0.001
NT-proBNP, pg/mL	204.9 [57.0, 968.3]	209.9 [55.9, 1010.0]	185.9 [62.2, 741.3]	181.7 [66.9, 696.8]	0.030	0.026	0.586	0.604
LVEF, n(%)	59.8 (11.8)	60.1 (11.7)	58.2 (12.2)	56.9 (12.7)	< 0.001	< 0.001	< 0.001	< 0.001
**Discharge medication use, n (%)**								
ACEI/ARB	79,073 (61.5)	65,917 (60.2)	10,179 (69.0)	2977 (70.2)	< 0.001	< 0.001	< 0.001	0.114
β-blocker	90,762 (70.6)	75,450 (68.9)	11,895 (80.6)	3417 (80.6)	< 0.001	< 0.001	< 0.001	0.975
Statins	106,451 (82.8)	88,835 (81.1)	13,714 (92.9)	3902 (92.1)	< 0.001	< 0.001	< 0.001	0.067
Aspirin	95,597 (74.4)	79,426 (72.5)	12,661 (85.8)	3510 (82.8)	< 0.001	< 0.001	< 0.001	< 0.001

*Abbreviation*: *ACEI/ARB* Angiotensin converting enzyme inhibitor/angiotensin receptor blockers, *CC* Coronary catheterization, *COPD* Chronic obstructive pulmonary disease, *eGFR* Estimated glomerular filtration rate, *HDLC* High density lipoprotein cholesterol, *hs-TnT* High sensitivity troponin T, *LDLC* Low density lipoprotein cholesterol, *LVEF* Left ventricular ejection fraction, *NT-proBNP* N-terminal pro-B-type natriuretic peptide, *SCr* Serum creatinine

### Study outcomes

**Fig. 2:**
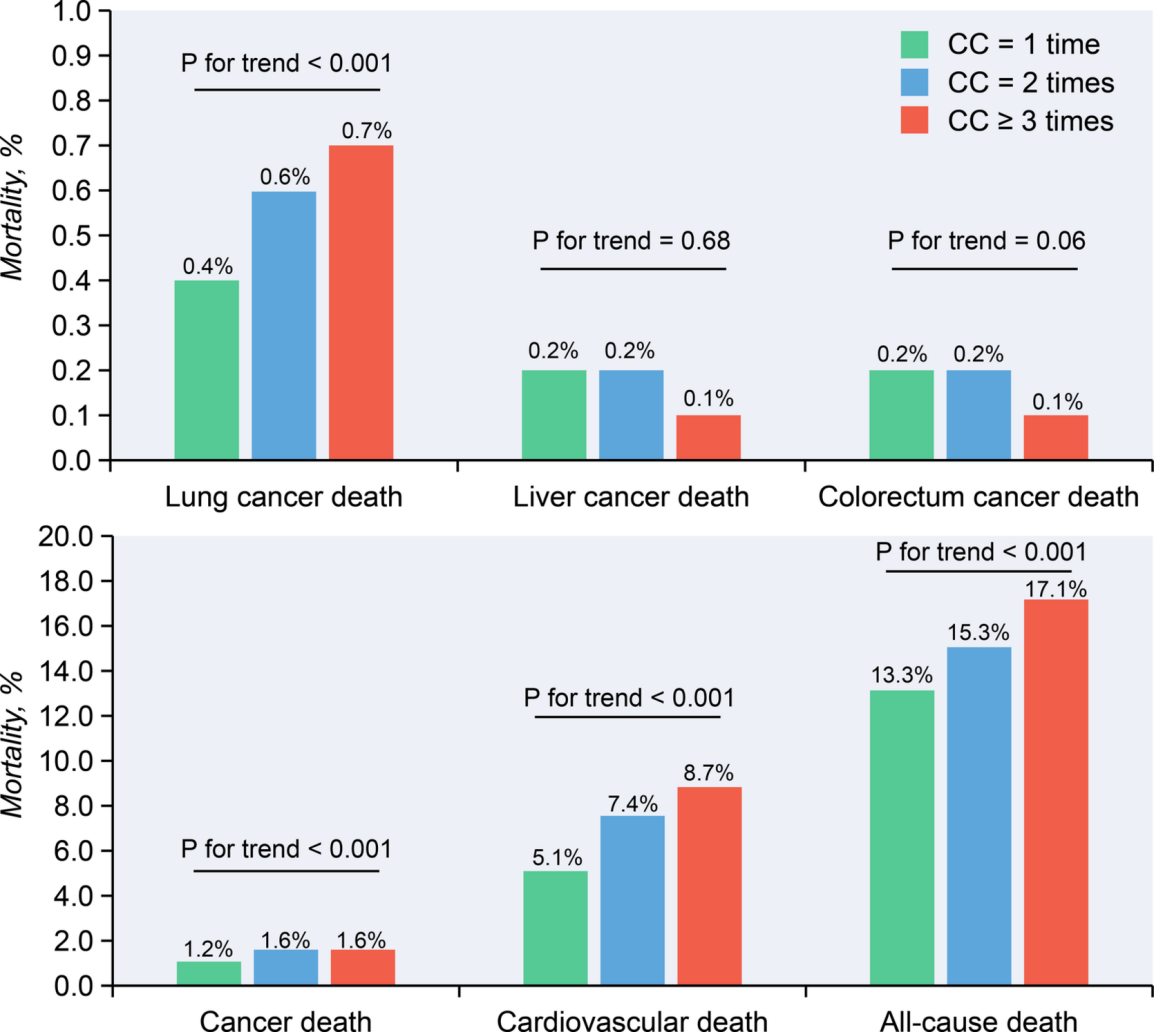
All-cause mortality and cumulative hazard for cardiovascular and cancer mortality of patients undergoing CAG

Among 136,495 hospitalized survivors with CC procedures, 18,656 patients (13.7%) died after discharge, of which 7,466 (5.5%) died of the cardiovascular disease and 617 (0.5%) died of lung cancer (Supplement Table 2). Proportional hazards assumption tests for the number of angiography and the radiology dose of angiography showed equal proportionality with mortality, therefore, we did not further consider the effects of time-varying exposure (Supplement Figs. 1 and 2). The patients with multiple CC procedures had higher lung cancer mortality (CC ≥ 3 times vs. 2 times vs. 1 time = 0.7%: 0.6%: 0.4%, *P for trend* < 0.001), but no significant difference was observed in liver cancer and colorectum cancer mortality. Moreover, patients receiving multiple CC procedures had higher all-cause mortality (CC ≥ 3 times vs. 2 times vs. 1 time = 17.1%: 15.3%: 13.3%, *P for trend* < 0.001), and cardiovascular mortality (CC ≥ 3 times vs. 2 times vs. 1 time = 8.7% vs. 7.4% vs. 5.1%, *P for trend* < 0.001) (Fig. 2).

After adjustments for confounders, compared with those receiving single CC procedures, patients with multiple CC procedures still had a higher risk of lung cancer mortality (CC 2 times: HR: 1.42 [95% CI, 1.13 to 1.78], *P* < 0.001; CC ≥ 3 times: HR: 1.64 [95% CI, 1.13 to 2.39], *P* < 0.05), all-cause mortality (CC 2 times: HR: 1.13 [95% CI, 1.08 to 1.19], *P* < 0.001; CC ≥ 3 times: HR: 1.33 [95% CI, 1.24 to 1.44], *P* < 0.001), and cardiovascular mortality (CC 2 times: HR: 1.35 [95% CI, 1.26 to 1.44], *P* < 0.001; CC ≥ 3 times: HR: 1.62 [95% CI, 1.46 to 1.80], *P* < 0.001), but this difference was not significant in liver cancer mortality, colorectum cancer mortality, and total cancer mortality (Table 2).

**Table 2:** The effect of radiation from coronary catheterization on mortality risk with radiation exposure represented with the cumulative number of coronary catheterization and cumulative effective dose after data complement

Endpoints	HR (95%CI)				
	**Cumulative number of procedures**			**Cumulative effective dose**	
	**CC = 1 time**	**CC = 2 times**	**CC ≥ 3 times**	≤ **15 mSv**	** > 15 mSv**
**Lung cancer mortality**					
Model 1	Ref	1.80 (1.45, 2.24)**	2.33 (1.61, 3.37)**	Ref	1.92 (1.55, 2.38)**
Model 2	Ref	1.39 (1.12, 1.73)**	1.55 (1.07, 2.25)*	Ref	1.48 (1.20, 1.84)**
Model 3	Ref	1.42 (1.13, 1.78)**	1.64 (1.13, 2.39)*	Ref	1.51 (1.21, 1.89)**
**Liver cancer mortality**					
Model 1	Ref	1.01 (0.66, 1.54)	0.71 (0.26, 1.90)	Ref	0.84 (0.53, 1.34)
Model 2	Ref	0.83 (0.54, 1.27)	0.52 (0.19, 1.39)	Ref	0.69 (0.43, 1.11)
Model 3	Ref	0.79 (0.51, 1.21)	0.49 (0.18, 1.33)	Ref	0.66 (0.41, 1.07)
**Colorectum cancer mortality**					
Model 1	Ref	1.49 (1.01, 2.21)*	1.32 (0.58, 3.00)	Ref	1.49 (0.99, 2.24)
Model 2	Ref	1.20 (0.80, 1.79)	0.92 (0.41, 2.09)	Ref	1.21 (0.80, 1.84)
Model 3	Ref	1.06 (0.71, 1.58)	0.80 (0.35, 1.81)	Ref	1.05 (0.69, 1.60)
**Total cancer mortality**					
Model 1	Ref	1.47 (1.28, 1.69)**	1.77 (1.39, 2.26)**	Ref	1.50 (1.30, 1.72)**
Model 2	Ref	1.17 (1.02, 1.35)*	1.23 (0.96, 1.57)	Ref	1.20 (1.04, 1.38)*
Model 3	Ref	1.13 (0.98, 1.31)	1.19 (0.93, 1.53)	Ref	1.15 (1.00, 1.33)*
**All-cause mortality**					
Model 1	Ref	1.31 (1.26, 1.37)**	1.70 (1.58, 1.83)**	Ref	1.35 (1.29, 1.41)**
Model 2	Ref	1.14 (1.09, 1.19)**	1.34 (1.25, 1.45)**	Ref	1.17 (1.12, 1.23)**
Model 3	Ref	1.13 (1.08, 1.19)**	1.33 (1.24, 1.44)**	Ref	1.16 (1.11, 1.22)**
**Cardiovascular mortality**					
Model 1	Ref	1.64 (1.54, 1.75)**	2.24 (2.02, 2.49)**	Ref	1.75 (1.65, 1.87)**
Model 2	Ref	1.38 (1.29, 1.47)**	1.69 (1.52, 1.88)**	Ref	1.49 (1.40, 1.59)**
Model 3	Ref	1.35 (1.26, 1.44)**	1.62 (1.46, 1.80)**	Ref	1.43 (1.34, 1.53)**

Model 1 was unadjusted; Model 2 was adjusted for sex and age; Model 3 was adjusted for sex, age, smoking history, coronary artery disease, hypertension, diabetes mellitus, congestive heart failure, chronic kidney disease, pulmonary infection, chronic obstructive pulmonary disease, atrial fibrillation, stroke, anemia, low density lipoprotein cholesterol, and high density lipoprotein cholesterol

^**^: *P* < 0.001; *P* < 0.05

Additional analyses were performed to show the mortality risk of high radiation exposure (> 15 mSv). We found that patients’ lung cancer mortality (HR: 1.51 [95% CI, 1.21 to 1.89], *P* < 0.001), total cancer mortality (HR: 1.15 [95% CI, 1.00 to 1.33],* P* < 0.05), all-cause mortality (HR: 1.16 [95% CI, 1.11 to 1.22],* P* < 0.001) and cardiovascular mortality (HR: 1.43 [95% CI, 1.34 to 1.53],* P* < 0.001) increased significantly with the increased time of CC procedures (Table 2).

**Fig. 3:**
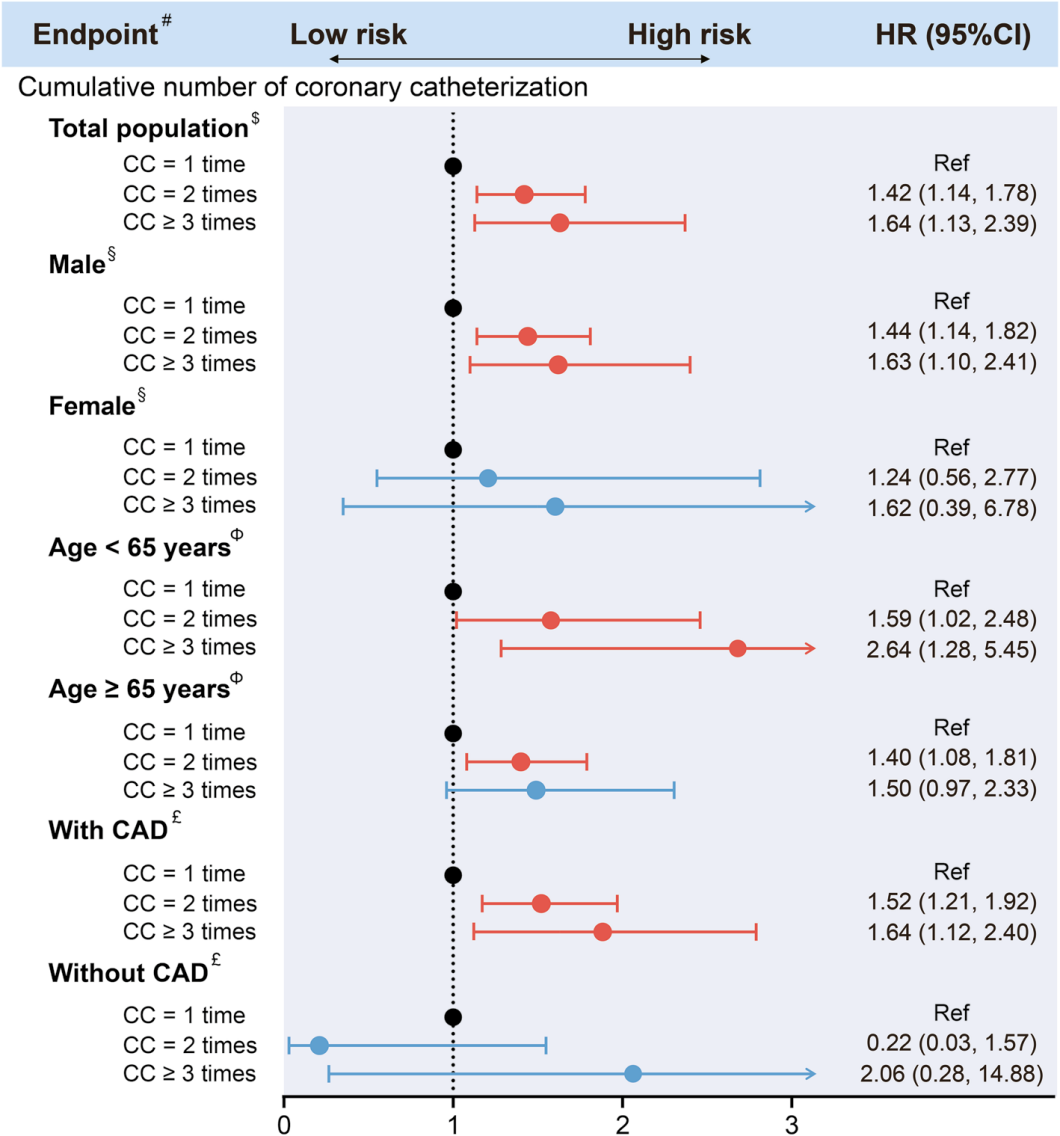
The effect of radiation from coronary catheterization on the mortality risk among different subgroups. Abbreviation: CC: coronary catheterization; CAD: coronary artery disease; HR: hazard radio #: lung cancer mortality $: Adjusted for sex, age, smoking history, coronary artery disease, hypertension, diabetes mellitus, congestive heart failure, chronic kidney disease, pulmonary infection, chronic obstructive pulmonary disease, atrial fibrillation, stroke, anemia, low density lipoprotein cholesterol, high density lipoprotein cholesterol §: Adjusted for age, smoking history, coronary artery disease, hypertension, diabetes mellitus, congestive heart failure, chronic kidney disease, pulmonary infection, chronic obstructive pulmonary disease, atrial fibrillation, stroke, anemia, low density lipoprotein cholesterol, high density lipoprotein cholesterol Φ: Adjusted for sex, smoking history, coronary artery disease, hypertension, diabetes mellitus, congestive heart failure, chronic kidney disease, pulmonary infection, chronic obstructive pulmonary disease, atrial fibrillation, stroke, anemia, low density lipoprotein cholesterol, high density lipoprotein cholesterol £: Adjusted for sex, age, smoking history, hypertension, diabetes mellitus, congestive heart failure, chronic kidney disease, pulmonary infection, chronic obstructive pulmonary disease, atrial fibrillation, stroke, anemia, low density lipoprotein cholesterol, high density lipoprotein cholesterol

Furthermore, the association between the radiation from CC procedures and lung cancer mortality were assessed among different subgroups. After adjustments for confounders, the number of CC procedures was still an independent risk factor of lung cancer mortality among male patients (CC 2 times: HR: 1.44 [95% CI, 1.14 to 1.82]; CC ≥ 3 times: HR: 1.63 [95% CI, 1.10 to 2.41]), patients aged < 65 years (CC 2 times: HR: 1.59 [95% CI, 1.02 to 2.48]; CC ≥ 3 times: HR: 2.64 [95% CI, 1.28 to 5.45]), and patients with CAD (CC 2 times: HR: 1.52 [95% CI, 1.21 to 1.92]; CC ≥ 3 times: HR: 1.64 [95% CI, 1.12 to 2.40]). (Fig. 3).

## Discussion

To our knowledge, this is the first multicenter study to evaluate the risk of lung cancer mortality induced by multiple times of coronary catheterization. In this study, we find that the risk of lung cancer mortality significantly increases with the times of coronary catheterization, while similar impact is not observed in other types of cancer. It suggests clinicians take effective measures to optimize disease management and reduce unnecessary subsequent radiation exposure due to repeated coronary catheterization for patients.

Our results show that multiple CC procedures are associated with higher risk of all-cause and cardiovascular mortality and lung-specific cancer mortality especially. In fact, with the development of medical technology, the proportion of imaging tests using is increasing year by year, which inevitably makes the subjects expose to a certain dose of radiation, and this trend is particularly evident among patients with known or suspected CAD [26]. Previous studies have reported that mean dose-area product values of CC procedures are 49.1 Gy cm^2^(effective dose ≈ 9.08 mSv), which is below the range of 10–50 mSv, the lowest dose acceptable for acute radiation exposure on human [27, 28]. Although the radiation dose of a single radiography is within the patient’s tolerable range, a study about the risk of cardiac imaging associated cancer in acute myocardial infarction patients have reported that with every 10 mSv of low-dose ionizing radiation, it can increase their risk of cancer by 3% and the perhaps mortality [11]. Indirectly, our study analyzes the risk of mortality in CC procedures patients and reaches the similar conclusions: with the increase of the number of CC procedures, the amount of radiation received by patients is also accumulating, and the risk of lung-specific cancer mortality as well as all-cause and cardiovascular mortality is increasing.

To our surprise, the risk of the total cancer mortality does not increase directly with the number of CC procedure, but after we analyze the top three specific cancers mortality, we find that lung cancer is particularly affected by imaging. We speculate that it may be because the radiation site is mainly located in the chest of the patients, leading to an increased risk of organ cells becoming cancerous in the corresponding area. Previous study has reported the top 3 most common types for global cancer-specific death were lung, liver and colorectum cancer [29], which is similar to the proportion of cancer deaths in our study. Given to the site of radiation exposure, the thyroid gland and the mammary gland in women beside the chest are also very sensitive to radiation, but the mortality of thyroid and breast cancer is low in our study, so they are not included in our analysis [30]. In addition, in our subgroup analysis, we find multiple CC procedures are associated with higher risk of lung cancer mortality among male patients, patients aged < 65 years, and patients with CAD. However, this association does not exist in patients without CAD. This may be because non-CAD patients primarily receive coronary angiography without further intervention treatment (such as PCI therapy), which results in much lower cumulative radiation doses for them compared to CAD patients (Supplement Fig. 3).

In our study, we find the risk of all-cause mortality, cardiovascular mortality and lung cancer mortality increases with the number of CC procedure, of which the lung cancer mortality increases particularly in the cancer-specific mortality. Although CC procedure is indispensable for the diagnosis of CAD, clinicians are supposed to considered the association between the radiation exposure from frequent CC procedures and patients’ risk of lung cancer and perhaps mortality before CC procedures, and weigh the benefit of CC procedures against mortality carefully, besides the adverse outcome related to their primary disease (e.g., coronary artery disease). In addition, it is necessary for clinicians to further manage the physical condition of patients after the CC procedure to reduce the risk of mortality, especially in patients with revascularization and in-stent restenosis.

### Limitations

This study has several limitations. Firstly, this study has limitations inherent in retrospective analysis, including data incompleteness, possible data inaccuracy, and selection bias. Secondly, we only analyze the association between exposure to radiation due to multiple coronary catheterization procedures and risk of cancer mortality, rather than cancer incidence. Nevertheless, few mortalities due to a secondary cause of cancer in this study are also counted as cancer mortalities during the data collection process, and the underestimation of cancer is minimized. Further studies are needed to validate the proportion and risk of mortality of CC procedure patients and improve their management to reduce the radiation exposure.

## Conclusion

In our study, multiple coronary catheterization procedures and higher radiation from coronary catheterization increases the risk of lung cancer mortality up to 40%-60%, while similar impact is not observed for other cancer types. Clinicians should improve the management of patients undergoing coronary catheterization to reduce subsequent radiation exposure to prevent the occurrence of lung cancer, and weigh the benefit of repeated procedure against mortality carefully.

### Supplementary Information

**Additional file 1: Supplement Table 1. **The effect of radiation from coronary catheterization on mortality risk with radiation exposure represented with the cumulative number of coronary catheterization and cumulative effective dose before data complement. **Supplement Table 2.** Mortality among patients undergoing coronary catheterization according to cumulative number of coronary catheterization and cumulative effective dose. **Supplement Figure 1. **Proportional Cox test was performed to assess the association between the number of Coronary Catheterization and lung cancer mortality. **Supplement Figure 2.** Proportional Cox test was performed to assess the association between the radiation dose and lung cancer mortality. **Supplement Figure 3**. Cumulative radiation dose from coronary catheterization procedure among patients with and without coronary artery disease. 

## Data Availability

The datasets used and/or analyzed during the current study available from the corresponding author on reasonable request.

## Abbreviations

CC: Coronary catheterization
CAG: Coronary angiography
CAD: Coronary artery disease
HT: Hypertension
DM: Diabetes mellitus
CHF: Congestive heart failure
CKD: Chronic kidney disease
