# Supplementary material for: Effect of cumulative radiation exposure from Coronary catheterization on lung cancer mortality
Source: BMC Cancer. 2023 Aug 15;23:757. doi: 10.1186/s12885-023-11231-4 (PMC10426196; doi:10.1186/s12885-023-11231-4)
Supplement: Supplementary file 1 — Additional file 1: Supplement Table 1. The effect of radiation from coronary catheterization on mortality risk with radiation exposure represented with the cumulative number of coronary catheterization and cumulative effective dose before data complement. Supplement Table 2. Mortality among patients undergoing coronary catheterization according to cumulative number of coronary catheterization and cumulative effective dose. Supplement Figure 1. Proportional Cox test was performed to assess the association between the number of Coronary Catheterization and lung cancer mortality. Supplement Figure 2. Proportional Cox test was performed to assess the association between the radiation dose and lung cancer mortality. Supplement Figure 3. Cumulative radiation dose from coronary catheterization procedure among patients with and without coronary artery disease. [file 12885_2023_11231_MOESM1_ESM.docx]

**Supplement Table 1. The effect of radiation from coronary catheterization on mortality risk with radiation exposure represented with the cumulative number of coronary catheterization and cumulative effective dose before data complement.**

| **Endpoints** | **HR (95%CI)** | | | | |
| --- | --- | --- | --- | --- | --- |
|  | **Cumulative number of procedures** | | | **Cumulative effective dose** | |
|  | **CC = 1 time** | **CC = 2 times** | **CC ≥ 3 times** | ≤ 15mSv | **> 15mSv** |
| **Lung cancer mortality** | | | | | |
| Model 1 | Ref | 1.80 (1.45, 2.24)** | 2.33 (1.61, 3.37)** | Ref | 1.92 (1.55, 2.38)** |
| Model 2 | Ref | 1.39 (1.12, 1.73)** | 1.55 (1.07, 2.25)* | Ref | 1.48 (1.20, 1.84)** |
| Model 3 | Ref | 1.54 (1.17, 2.02)* | 1.71 (1.09, 2.68)* | Ref | 1.52 (1.15, 2.01)** |
| **Liver cancer mortality** | | | | | |
| Model 1 | Ref | 1.01 (0.66, 1.54) | 0.71 (0.26, 1.90) | Ref | 0.84 (0.53, 1.34) |
| Model 2 | Ref | 0.83 (0.54, 1.27) | 0.52 (0.19, 1.39) | Ref | 0.69 (0.43, 1.11) |
| Model 3 | Ref | 0.77 (0.46, 1.28) | 0.46 (0.15, 1.47) | Ref | 0.59 (0.33, 1.06) |
| **Colorectum cancer mortality** | | | | | |
| Model 1 | Ref | 1.49 (1.01, 2.21)* | 1.32 (0.58, 3.00) | Ref | 1.49 (0.99, 2.24) |
| Model 2 | Ref | 1.20 (0.80, 1.79) | 0.92 (0.41, 2.09) | Ref | 1.21 (0.80, 1.84) |
| Model 3 | Ref | 1.12 (0.70, 1.78) | 0.51 (0.16, 1.61) | Ref | 1.06 (0.65, 1.72) |
| **Total cancer mortality** | | | | | |
| Model 1 | Ref | 1.47 (1.28, 1.69)** | 1.77 (1.39, 2.26)** | Ref | 1.50 (1.30, 1.72)** |
| Model 2 | Ref | 1.17 (1.02, 1.35)* | 1.23 (0.96, 1.57) | Ref | 1.20 (1.04, 1.38)* |
| Model 3 | Ref | 1.13 (0.95, 1.34) | 1.04 (0.77, 1.41) | Ref | 1.08 (0.90, 1.29) |
| **All-cause mortality** | | | | | |
| Model 1 | Ref | 1.31 (1.26, 1.37)** | 1.70 (1.58, 1.83)** | Ref | 1.35 (1.29, 1.41)** |
| Model 2 | Ref | 1.14 (1.09, 1.19)** | 1.34 (1.25, 1.45)** | Ref | 1.17 (1.12, 1.23)** |
| Model 3 | Ref | 1.17 (1.10, 1.23)** | 1.32 (1.20, 1.44)** | Ref | 1.16 (1.10, 1.23)** |
| **Cardiovascular mortality** | | | | | |
| Model 1 | Ref | 1.64 (1.54, 1.75)** | 2.24 (2.02, 2.49)** | Ref | 1.75 (1.65, 1.87)** |
| Model 2 | Ref | 1.38 (1.29, 1.47)** | 1.69 (1.52, 1.88)** | Ref | 1.49 (1.40, 1.59)** |
| Model 3 | Ref | 1.40 (1.29, 1.51)** | 1.50 (1.32, 1.71)** | Ref | 1.41 (1.30, 1.52)** |

Model 1 was unadjusted; Model 2 was adjusted for sex and age; Model 3 was adjusted for sex, age, smoking history, coronary artery disease, hypertension, diabetes mellitus, congestive heart failure, chronic kidney disease, pulmonary infection, chronic obstructive pulmonary disease, atrial fibrillation, stroke, anemia, low density lipoprotein cholesterol, and high density lipoprotein cholesterol.

**: *P* <0.001; *P* <0.05.

**Supplement Table 2. Mortality among patients undergoing coronary catheterization according to cumulative number of coronary catheterization and cumulative effective dose.**

| **Endpoints** | **Events (*n, %*)** | | | | | |
| --- | --- | --- | --- | --- | --- | --- |
|  | **Overall** | **Cumulative number of procedures** | | | **Cumulative effective dose** | |
|  |  | **CC time = 1** | **CC time = 2** | **CC time ≥ 3** | ≤ **15mSv** | **> 15mSv** |
| **Lung cancer mortality** | 617 (0.5) | 489 (0.4) | 98 (0.6) | 30 (0.7) | 517 (0.4) | 100 (0.6) |
| **Liver cancer mortality** | 239 (0.2) | 211 (0.2) | 24 (0.2) | 4 (0.1) | 220 (0.2) | 19 (0.1) |
| **Colorectum cancer mortality** | 211 (0.2) | 176 (0.2) | 29 (0.2) | 6 (0.1) | 184 (0.2) | 27 (0.2) |
| **Total cancer mortality** | 1741 (1.3) | 1438 (1.2) | 236 (1.6) | 67 (1.6) | 1513 (1.3) | 228 (1.4) |
| **All-cause mortality** | 18656 (13.7) | 15595 (13.3) | 2324 (15.3) | 737 (17.1) | 16364 (13.6) | 2292 (14.5) |
| **Cardiovascular mortality** | 7466 (5.5) | 5975 (5.1) | 1117 (7.4) | 374 (8.7) | 6305 (5.2) | 1161 (7.3) |

Abbreviation: CC: coronary catheterization

**Supplement figure 1: Proportional Cox test was performed to assess the association between the number of Coronary Catheterization and lung cancer mortality**


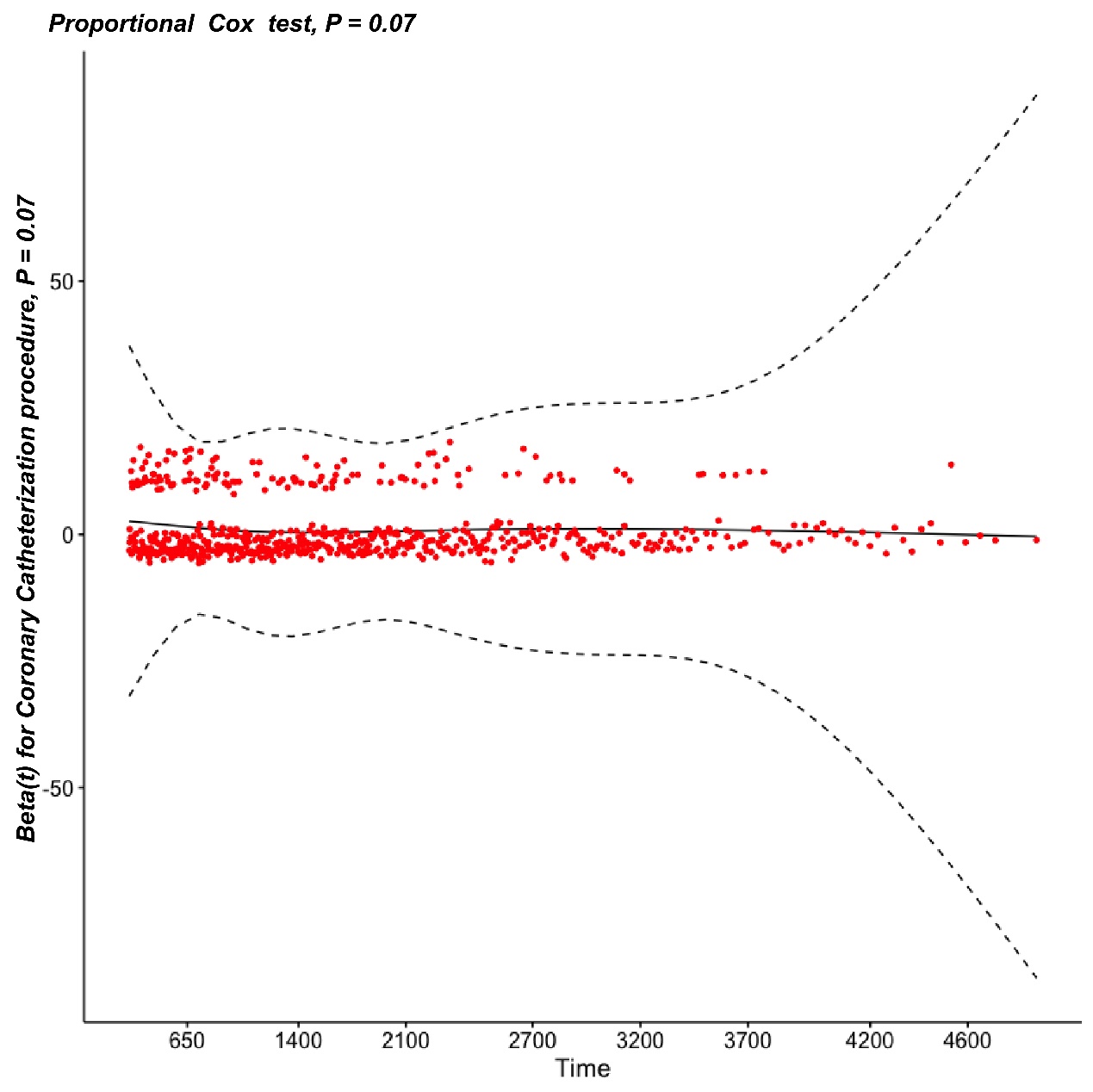


Adjusted for sex, age, smoking history, coronary artery disease, hypertension, diabetes mellitus, congestive heart failure, chronic kidney disease, pulmonary infection, chronic obstructive pulmonary disease, atrial fibrillation, stroke, anemia, low density lipoprotein cholesterol, and high density lipoprotein cholesterol.

**Supplement figure 2: Proportional Cox test was performed to assess the association between the radiation dose and lung cancer mortality**


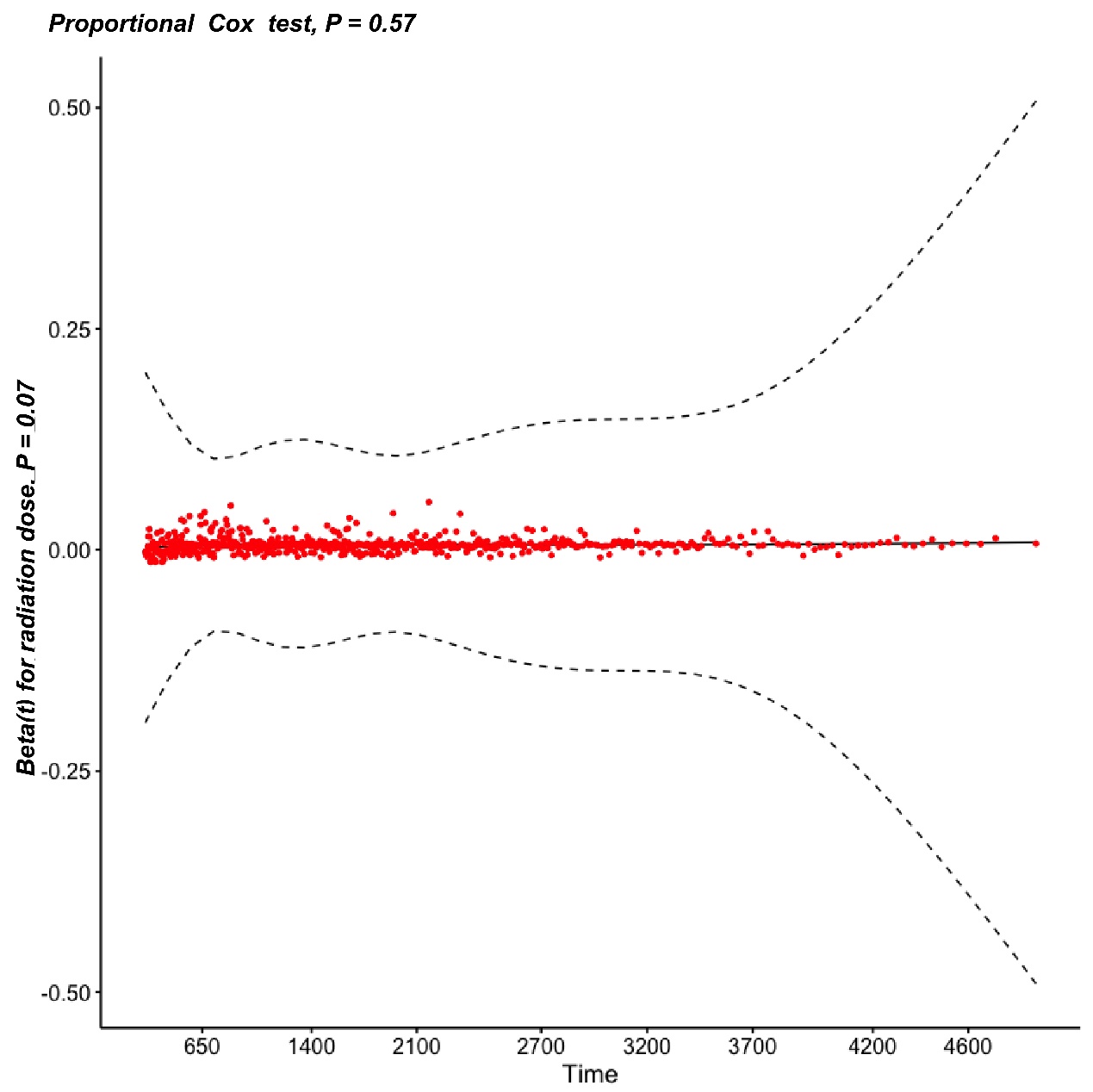


Adjusted for sex, age, smoking history, coronary artery disease, hypertension, diabetes mellitus, congestive heart failure, chronic kidney disease, pulmonary infection, chronic obstructive pulmonary disease, atrial fibrillation, stroke, anemia, low density lipoprotein cholesterol, and high density lipoprotein cholesterol.

**Supplement figure 3: Cumulative radiation dose from coronary catheterization procedure among patients with and without coronary artery disease**


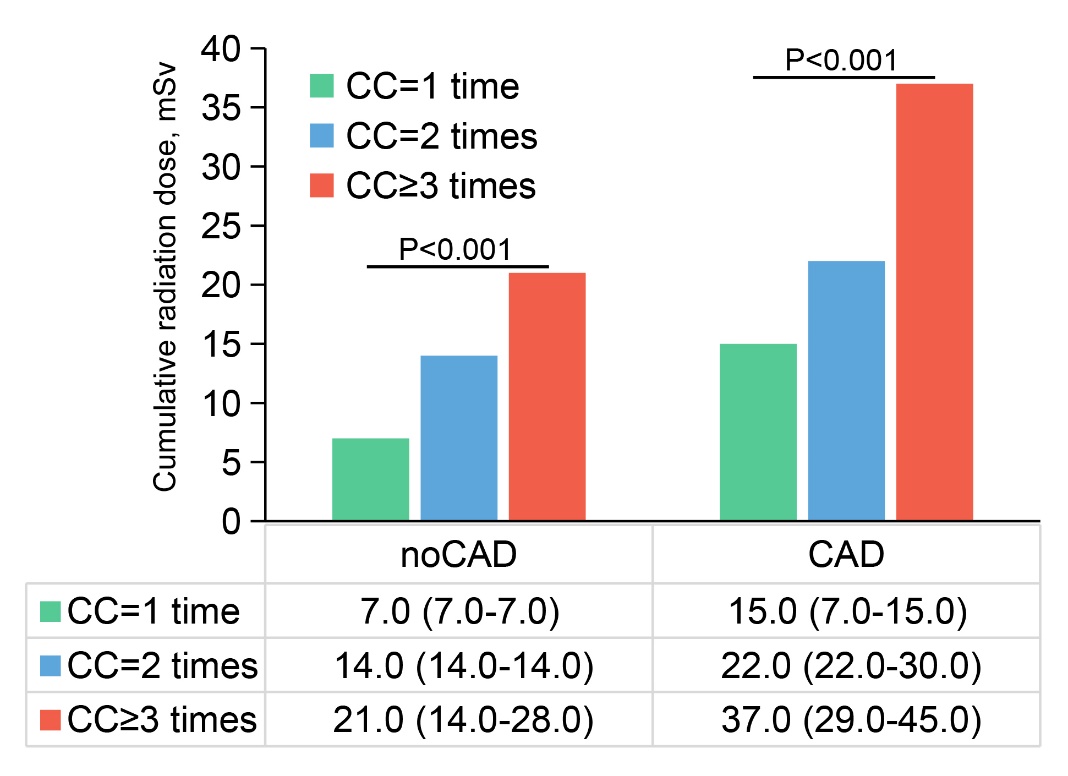


Cumulative radiation dose was presented as median (IQR).
